# Toward the Next Generation of In Silico Modeling of Dynamic Host-Microbiota Interactions in the Skin

**DOI:** 10.1016/j.xjidi.2025.100385

**Published:** 2025-05-14

**Authors:** Jamie Lee, Hiu Lam Athena Wu, Ahmad A. Mannan, Yuumi Nakamura, Masayuki Amagai, Alan D. Irvine, Reiko J. Tanaka

**Affiliations:** 1 Department of Bioengineering, Imperial College London, London, United Kingdom; 2 Cutaneous Allergy and Host Defense, Immunology Frontier Research Center, The University of Osaka, Osaka, Japan; 3 Department of Dermatology, Keio University School of Medicine, Tokyo, Japan; 4 Clinical Medicine, Trinity College Dublin, Dublin, Ireland

**Keywords:** In silico modeling, skin disease, skin microbiome, time-course data, validation

## Abstract

Understanding how the skin microbiota contributes to skin health and disease requires knowledge of the dynamic interactions between the skin and its resident microbes. In silico modeling complements in vivo and in vitro experiments by enabling a systems-level understanding of dynamic skin-microbiota interactions. However, the number of published in silico skin microbiota models remains limited. This paper provides the first comprehensive exploration of in silico skin microbiota modeling. We identify current challenges, learn from leading experimental validation approaches adopted in in silico gut microbiota research, and propose ways to enhance the predictive power of in silico skin microbiota models.

## Introduction

The human skin microbiota plays a crucial role in maintaining our skin’s health (Byrd et al, 2018; Flowers and Grice, 2020; Naik et al, 2015; Paller et al, 2019). It supports the cutaneous innate immune defence by activating local immune cells (Belkaid and Segre, 2014; Cogen et al, 2010; Lai et al, 2010; Nakatsuji et al, 2017) and creating a pathogen-antagonising environment (Brown et al, 2020; Hülpüsch et al, 2024; Iwase et al, 2010; Paharik et al, 2017; Parlet et al, 2019; O’Sullivan et al, 2019). An alteration to the “healthy” skin microbiota (known as dysbiosis) is characterized by a change in microbial composition and reduced diversity, and has been associated with exacerbating symptoms in skin diseases such as atopic dermatitis (AD) (Byrd et al, 2017; Darlenski et al, 2021; Kobayashi et al, 2015), acne vulgaris (Dessinioti and Katsambas, 2024; Huang et al, 2023; Lee et al, 2019), and psoriasis (Langan et al, 2019; Yan et al, 2017). Although renormalization of the microbiota to a higher diversity through treatment (eg, corticosteroids [Nørreslet et al, 2025], diluted bleach baths [Khadka et al, 2021], biologics [Callewaert et al, 2020], and bacteriotherapy [Myles et al, 2018; Nakatsuji et al, 2021a, 2021b]) appears to correlate with a stable recovery to healthier skin conditions, developing the rationale behind which microbes (probiotics) and nutrients (prebiotics) to add, how much, and when is still hindered by a currently limited understanding of skin-microbiota interactions and how these shape the dynamic rise and fall of microbial populations.

To date, culture-based experimental efforts to unravel skin-microbiota interactions and their dynamic responses to perturbations have mainly been conducted on 1 or 2 skin microbes grown in vitro in well plates (Nakatsuji et al, 2017), human organotypic skin models (Kohda et al, 2021; Rikken et al, 2023), and on in vivo mouse models (Almoughrabie et al, 2023; Burian et al, 2017; Williams et al, 2019; Zheng et al, 2022). Although these studies are crucial for uncovering the impact of one microbe on another or the skin, studying microbial interactions experimentally at the microbiota level remains challenging owing to the exponential increase in interdependencies and crosstalk in cellular signaling as the microbial community increases in size (Widder et al, 2016). In addition, despite growing evidence of substantial phenotypic variation within strains, in vitro microbiota studies often neglect how these strain-level differences influence microbial function and community behavior (Nakamura et al, 2020). This raises concerns about whether observed microbial interactions and dynamics are truly species-level responses or merely strain-specific phenomena.

On the other hand, time-course 16S ribosomal RNA and whole-genome shotgun sequencing data can provide a comprehensive microbiota-level overview of the diverse microbial species present in the skin, particularly regarding the temporal stability of the skin microbiota and how its composition changes across skin conditions and body sites (Byrd et al, 2017; Oh et al, 2016; Shi et al, 2016). However, insights derived are primarily correlational, and mechanistic causal relationships cannot be deduced from such data alone.

Integrating in silico modeling with experimental methods can help achieve a systems-level overview of the dynamic interactions within skin microbial communities. In silico modeling is a computational approach where our understanding of dynamic processes and interactions are described using mathematical equations. Simulations using in silico models can help to better understand disease pathogenesis and guide treatment design by exploring hypothetical scenarios often too complex to test in wet-laboratory experiments or clinical studies. For instance, in silico models can predict how specific bacterial strains within microbial communities could respond to variations in the skin environment or bacteriotherapy, providing insights that would require ample time and resources if studied only by in vitro experiments. Furthermore, through simulating individualized microbiota responses based on patient-specific characteristics, in silico modeling could help address the complexity of host-microbiota interactions and offer personalized insights into disease progression and treatment efficacy in diseases such as AD, where patients exhibit significant phenotypic variations (Langan et al, 2020). This integrated approach can provide a more structured framework for investigating causal relationships and predicting therapeutic outcomes, helping bridge critical gaps in conventional microbiome research methods.

The development of in silico models typically follows an iterative “learn-build-predict-validate” cycle. To begin with, we learn from experimental data. Empirical data are collected through tailored experiments or a literature review to assemble hypothetical interaction networks between microbes and host components. Next, an in silico model is built for the microbial community of interest, where model parameters represent biological relationships between microbes, host factors, and other components modeled (eg, microbial growth rates, strengths of interactions, production and consumption rates of metabolites, cell death rates). Depending on the preliminary model assumptions, these biological relationships can be represented using different mathematical formulations, for example, ordinary differential equations (ODEs), genome-scale metabolic models (GEMs), and agent-based models (ABMs). ODEs are commonly used to describe how microbial populations and host responses change over time and typically assume a well-mixed environment, whereas GEMs offer a comprehensive view of metabolic interactions on a cellular level, either within microbes when used on their own or between several microbes when the GEMs are connected through exchange reactions. ABMs, on the other hand, simulate spatiotemporal dynamics of individual entities (ie, “agents”) to capture spatial structure and heterogeneity in microbial communities. Table 1 summarizes the studies reviewed in this paper, grouped by the 3 types of in silico models discussed. The basis of these in silico models has been extensively explored in other review papers (Colarusso et al, 2021; Gonze et al, 2018; Kumar et al, 2019; Nagarajan et al, 2022; Qian et al, 2021).

**Table 1:** Summary of In Silico Skin and Gut Microbiota Studies Reviewed in this Paper

Model Type	References	
	In Silico Skin Microbiota Studies	In Silico Gut Microbiota Studies
ODE	Lee et al, 2024; Miyano et al, 2022; Nakaoka et al, 2016; Thibault Greugny et al, 2024	Brunner and Chia, 2019, 2024; Bucci et al, 2016; Connors et al, 2023; Coyte et al, 2015; D’hoe et al, 2018; Evdokimova et al, 2022; Guittar et al, 2021; Ho et al, 2024; Hromada et al, 2021; Ke et al, 2023; Kettle et al, 2018; Marino et al, 2014; Pinto et al, 2017; Rao et al, 2021; Shibasaki and Mitri, 2023; Stein et al, 2018, 2013; Venturelli et al, 2018; Wang et al, 2020; Wiles et al, 2016; Xiong et al, 2021
GEM	Kim et al, 2023	Basile et al, 2023; Bauer et al, 2017; Bidkhori and Shoaie, 2024; Brunner and Chia, 2024; da Silva et al, 2024; Diener et al, 2020; Greenhalgh et al, 2019; Harcombe et al, 2014; Heinken and Thiele, 2015a, 2015b; Heinken et al, 2019; Henson and Phalak, 2017, 2018; Hertel et al, 2021, 2023; Hirmas et al, 2022; Hoek and Merks, 2017; Iyengar and Perry, 2024; Khandelwal et al, 2013; Lambert et al, 2024; Noecker et al, 2019; Salahshouri et al, 2021; Shoaie et al, 2013, 2015; Steinway et al, 2015; Thiele et al, 2020; Velasco-Álvarez et al, 2023; Versluis et al, 2024; Zelezniak et al, 2015
ABM	Montgomery et al, 2024	Bauer et al, 2017; Coyte et al, 2015; Holmes et al, 2017; Schluter and Foster, 2012; Seal et al, 2011; Valiei et al, 2024

Abbreviations: ABM, agent-based model; GEM, genome-scale metabolic model; ODE, ordinary differential equation.

The final step of the model-building stage involves determining parameter values by fitting the constructed model to gathered data. This process quantifies the biological mechanisms and relationships between modeled components in the microbial community of interest. Such models can then be used to make predictions. For instance, we can simulate the dynamic response and long-term outcomes of the microbial community to perturbations (eg, the addition of bacteriotherapy, infection by pathogens, or changes in the skin environment), thereby contributing to our understanding of skin-microbiota interactions and the rational design of therapies. Finally, performing validation experiments can confirm the robustness of in silico model predictions. Discrepancies between model predictions and observations from validation experiments would indicate a need to revisit the model assumptions or missing biological knowledge in the in silico representation, and new data from validation experiments can then serve as the starting point of the next “learn-build-predict-validate” cycle.

Applications of in silico modeling to skin microbiota research is an emerging field, where only 6 papers have been published so far (Kim et al, 2023; Lee et al, 2024; Miyano et al, 2022; Montgomery et al, 2024; Nakaoka et al, 2016; Thibault Greugny et al, 2024), 5 of which were published within the last 3 years (Kim et al, 2023; Lee et al, 2024; Miyano et al, 2022; Montgomery et al, 2024; Thibault Greugny et al, 2024). This review aims to provide the first comprehensive exploration of the field to highlight the utility of in silico skin microbiota models, specifically by reviewing the current research landscape, identifying existing challenges, and proposing potential solutions to advance the field. Ultimately, we envision that in silico modeling of the skin microbiota will facilitate a systems-level understanding of the factors driving skin microbiota dynamics.

## Results

### Current research landscape of in silico host-microbe and microbe-microbe models of the skin

The literature review of published in silico models of the skin microbiota identified 6 relevant studies, details of which are summarized in Table 2. Four of them describe the interactions between 2 microbes and the skin by modeling population-level dynamics (Lee et al, 2024; Miyano et al, 2022; Nakaoka et al, 2016; Thibault Greugny et al, 2024), 1 study focused on the metabolic pathways within a bacterium (Kim et al, 2023), and 1 study described the spatiotemporal dynamics of 3 bacterial genera commonly found on human skin (Montgomery et al, 2024).

**Table 2:** In Silico Models of Host-Microbe and Microbe-Microbe Interactions in the Skin

Study	Model Type	Skin Condition	Key Research Question	Key In Silico Predictions	Have Model Predictions Been Validated Yet?
Nakaoka et al, 2016	ODE	General (skin inflammation)	What are the key mechanisms regulating the shift in beneficial and harmful bacterial populations in the epidermis?	• In immune cells, transcription factors activate proteases, playing a crucial role in regulating bacterial persistence. • Varying cytokine clearance rates lead to 3 phenotypes: (i) persistence of harmful bacteria, (ii) persistence of beneficial bacteria, or (iii) coexistence of both.	No
Miyano et al, 2022	ODE	AD	Why do SA-targeted treatments for AD show varying efficacies in clinical trials?	• The antimicrobial action of bacteriotherapies worsens AD severity by impairing CoNS’ ability to inhibit SA’s quorum sensing and virulence factor production. • Selectively killing SA by 10^3^ CFU/cm^2^ achieved an efficacy comparable to dupilumab. • Maximum efficacy was achieved by combining SA-killing with dupilumab, outperforming either treatment alone.	No
Kim et al, 2023	GEM	Acne vulgaris	Which metabolic pathways drive *C acnes* pathogenicity in acne vulgaris?	• In glycerol-rich conditions, *C* *acnes* overproduces propionate, potentially exacerbating inflammation in acne vulgaris. • The “Wood-Werkman cycle” is strongly activated in acne vulgaris skin, driving pathogenicity in sebum-rich conditions. • Two enzymes were identified as promising targets for selectively inhibiting *C* *acnes* growth, offering a new avenue for acne vulgaris treatment.	No
Thibault Greugny et al, 2024	ODE	General (skin microbiota)	What are the key mechanisms driving the dominance of pathogenic populations over commensals in the skin microbiota?	• An elevation in skin pH can lead to the dominance of pathogens. • Host AMPs influence the balance between pathogens and commensals non-linearly, with higher levels potentially encouraging pathogen dominance.	No
Lee et al, 2024	ODE	AD	What are the key mechanisms driving skin damage by SA and SE in AD lesions?	• Strong inhibition of SA and SE growth by the skin, and fast skin turnover and repair are key mechanisms supporting an undamaged skin state in AD. • Continuous SA and SE attenuation, combined with temporary SA-Killing, was identified as a promising treatment strategy.	No
Montgomery et al, 2024	ABM	General (skin microbiota)	How feasible is *Bsubtilis* as a topical drug delivery system?	• When introduced to the native skin microbiota, *B* *subtilis* survived for approximately half a day before being rapidly eliminated. • Providing a carbon source specific to *B* *subtilis* (0.37 mmol/cm^2^ of malate) extended its survival time in the native skin microbiota from approximately half a day to a day. • Antibiotic treatment enabled *B* *subtilis’* long-term survival but led to the eradication of *Acinetobacter* populations.	Yes

Abbreviations: ABM, agent-based model; AD, atopic dermatitis; AMP, antimicrobial peptide; CFU, colony forming unit; CoNS, coagulase-negative *Staphylococci*; GEM, genome-scale metabolic model; ODE, ordinary differential equation; SA, *Staphylococcus aureus*; SE, *Staphylococcus epidermidis*.

Thibault Greugny et al (2024) and Nakaoka et al (2016) studied how the balance between populations of good and bad bacteria (of unspecified microbes) is affected by changes in the skin environment (eg, antimicrobial peptide [AMP] concentration, pH, and immune response). The Thibault Greugny et al (2024) model predicted, for example, that an elevation in skin pH can encourage the long-term persistence of pathogens, highlighting the importance of skin acidification in maintaining healthy skin function. The Nakaoka et al (2016) model suggested that higher rates of cytokine clearance can eliminate harmful bacteria, providing an experimentally testable hypothesis.

Miyano et al (2022) and Lee et al (2024) developed in silico models that describe key microbial interactions in AD lesions, specifically between a pathogen, *Staphylococcus aureus* (SA), and commensal species, coagulase-negative *Staphylococci* (CoNS). Miyano et al (2022) developed a quantitative systems pharmacology (QSP) model parameterized by clinical data from 3 studies to identify potential explanations for conflicting efficacies observed in SA-targeted treatments. Their QSP model revealed that SA-targeted treatments can inadvertently kill CoNS, leading to worsened AD symptoms due to the reduced ability of CoNS to inhibit SA virulence at a population level. Simulations predicted that selectively killing SA (but not CoNS) matched dupilumab’s efficacy, and combining SA-killing treatments with dupilumab enhanced treatment outcomes, presenting a testable treatment regimen. While Miyano et al (2022) considered a nondamaging CoNS, Lee et al (2024) modeled skin-damaging strains of both SA and *Staphylococcus epidermidis* (SE) to understand the key mechanisms driving skin damage in AD lesions. The in silico model simulations suggested that specifically killing SA could cause SE strains to switch to a skin-damaging phenotype and that an enhancement of skin barrier function to attenuate the growths of SA and SE was necessary for a treatment specifically killing SA to enable the re-establishment of a high skin barrier integrity.

Although the models discussed earlier describe the population-level dynamics of multiple microbes through ODEs (Lee et al, 2024; Miyano et al, 2022; Nakaoka et al, 2016; Thibault Greugny et al, 2024), Kim et al (2023) developed a GEM of *Cutibacterium* *acnes*’ genome-scale metabolism consisting of 1510 reactions. The model recapitulated the bacterium’s pathogenic traits in acne vulgaris through its synthesis of propionate and acetate. Model analysis found that the “Wood-Werkman” cycle was strongly activated in sebum-rich environments, leading to an overproduction of propionate, which may play a role in the pathogenesis of acne vulgaris. The model was then used to identify potential metabolic targets to impair *C* *acnes’* pathogenesis, contributing toward the development of acne vulgaris treatments.

Montgomery et al (2024) recently developed an ABM to simulate spatiotemporal and heterogenous behaviors in microbial communities, assessing the feasibility of using an engineered microbe, *Bacillus* *subtilis*, to deliver topical skin treatments for diseases such as psoriasis and AD. They modeled the growth and death of 3 common microbial genera on the skin (*Staphylococcus*, *Corynebacteria*, and *Acinetobacter*) through mathematical descriptions of their carbon consumption of various nutrient sources on the basis of literature data. Model simulations suggested that *B* *subtilis* can survive for half a day before being rapidly cleared when added to the 3-genera microbial community, and that its survival could be prolonged by introduction of antibiotics and *B* *subtilis-*specific nutrient sources. These findings were subsequently confirmed by Montgomery et al (2024) using tailored ex vivo (pig skin), in vitro (human skin tissue), and in vivo (murine) experiments, demonstrating the promise of in silico modeling in guiding the design of microbiota-based treatments.

Altogether, these in silico models can inform the rational design of probiotics and prebiotics by predicting how the skin microbiota can be manipulated to inhibit the pathogenic traits of microbes (Kim et al, 2023; Lee et al, 2024; Miyano et al, 2022; Nakaoka et al, 2016; Thibault Greugny et al, 2024) and how prebiotic strategies can prolong probiotic survival (Montgomery et al, 2024) in the native skin environment.

### Key gaps between in silico modeling of the skin microbiota and that of the gut microbiota

**Figure 1:**
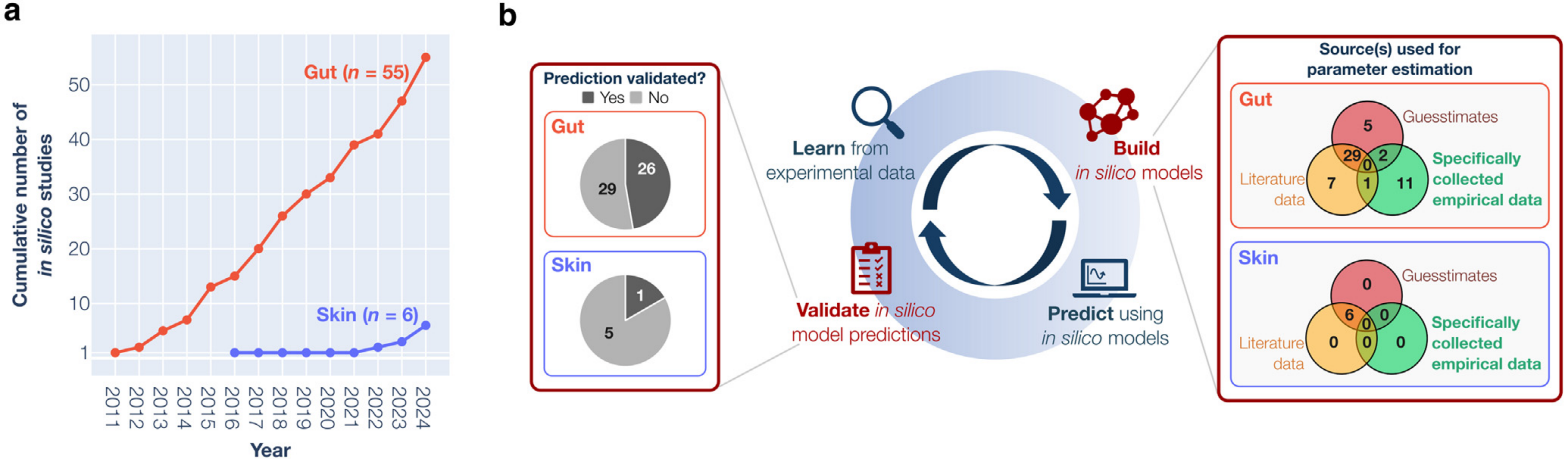
**Comparison between the in silico skin and gut microbiota models reviewed.** (**a**) Trends in the number of in silico gut (red) and skin (blue) models over the years. (**b**) Pain points (red text) in the “learn-build-predict-validate” cycle of in silico skin microbiota modeling. The box on the left summarizes the number of studies that validated model predictions, whereas the box on the right categorizes studies on the basis of how model parameters were derived, whether using guesstimates, literature data, or specifically collected empirical data.

Although in silico research efforts on the skin microbiota and its role in health and disease are on the rise, the research landscape is still relatively underdeveloped compared with that of in silico research of other human microbiota sites (NIH Human Microbiome Portfolio Analysis Team et al, 2019). To learn from and help identify how to accelerate in silico skin microbiota research, we conducted a literature review of published in silico models of the gut microbiota, one of the most extensively studied areas of the human microbiota (NIH Human Microbiome Portfolio Analysis Team et al, 2019). We identified 55 studies on the basis of our search method (Figure 1a and Materials and Methods).

In contrast to the skin, in silico gut microbiota models typically represent more intricate microbial communities, frequently encompassing over 10 microbes (Bucci et al, 2016; Connors et al, 2023; Coyte et al, 2015; Heinken and Thiele, 2015a, 2015b; Henson and Phalak, 2018; Ho et al, 2024; Hromada et al, 2021; Noecker et al, 2019; Rao et al, 2021; Shoaie et al, 2015; Stein et al, 2013, 2018; Thiele et al, 2020; Venturelli et al, 2018). These models have been employed to reveal how interactions between microbes may influence the function of the microbial community and impact its stability (Bauer et al, 2017; Coyte et al, 2015; D’hoe et al, 2018; Harcombe et al, 2014; Henson and Phalak, 2018; Ho et al, 2024; Pinto et al, 2017). Similar questions around community behavior are yet to be addressed for the skin microbiota. The models we reviewed only capture the interactions between a maximum of 3 microbes and some host components (Montgomery et al, 2024).

Our analysis of in silico gut microbiota models hinted at 2 key bottlenecks in advancing applications of in silico modeling to skin microbiota research: (i) the lack of fit-for-purpose empirical data available during the model development process (Figure 1b, build) and (ii) the lack of prediction validation (Figure 1b, validate).

Fourteen of 55 in silico gut microbiota models were developed using empirical data specifically collected for model development (Table 3 and Figure 1b, top-right). However, none of the in silico skin microbiota models did. Instead, they were informed by guesstimates (ie, parameter values assigned on the basis of educated guesses rather than precise experimental data) and secondary literature data (Figure 1b, bottom-right). Data specifically collected from tailored experiments play a crucial role in the development of in silico models because they can help refine in silico model parameters and structure, which should, in turn, improve model performance in subsequent predictions. Model parameters obtained by fitting to tailored experimental data are expected to be more context specific and hence less error prone than those derived from guesstimates or an assumption-based contextual translation of literature data.

**Table 3:** Summary of In Silico Gut Microbiota Studies that Incorporated Specifically Collected Empirical Data as Part of their Model-Building Process or Experimentally Validated their Model Predictions

Categories	References
Studies where empirical data were specifically collected for model development	Basile et al, 2023; Bucci et al, 2016; Connors et al, 2023; D’hoe et al, 2018; Evdokimova et al, 2022; Greenhalgh et al, 2019; Ho et al, 2024; Hromada et al, 2021; Marino et al, 2014; Pinto et al, 2017; Rao et al, 2021; Stein et al, 2018; Venturelli et al, 2018; Wiles et al, 2016
Studies where model predictions were experimentally validated	Bauer et al, 2017; Bucci et al, 2016; Connors et al, 2023; D’hoe et al, 2018; Diener et al, 2020; Evdokimova et al, 2022; Greenhalgh et al, 2019; Harcombe et al, 2014; Hertel et al, 2021; Hirmas et al, 2022; Ho et al, 2024; Holmes et al, 2017; Hromada et al, 2021; Ke et al, 2023; Marino et al, 2014; Pinto et al, 2017; Rao et al, 2021; Seal et al, 2011; Shoaie et al, 2013, 2015; Stein et al, 2013, 2018; Steinway et al, 2015; Thiele et al, 2020; Venturelli et al, 2018; Wiles et al, 2016

Furthermore, validation experiments are currently lacking to confirm predictions made using in silico skin microbiota models. Of the 55 in silico gut microbiota studies reviewed, 26 of the studies have their model predictions experimentally validated (Table 3 and Figure 1b, top-left). In contrast, only 1 skin microbiota study reported validation results of their model predictions (Montgomery et al, 2024) (Figure 1b, bottom-left).

The iterative “learn-build-predict-validate” cycle between model validation and development (Figure 1b) is especially critical for clinical applications and therapy design because it allows a reality check to confirm our mechanistic understanding of biological processes occurring in the skin microbiota. Without validation, model predictions are left as plausible outcomes, and the reliability and utility of in silico skin microbiota models remain uncertain.

## Discussion

On the basis of our literature review of in silico gut microbiota models and their validation methods, we propose ways to gather tailored empirical data and validate predictions from in silico skin microbiota models using current in vivo and in vitro experiments to advance this research area.

### Three ideal characteristics of data for experimental validation

To effectively validate in silico model predictions, the ideal experimental data collected should (i) be time-course data, (ii) be collected under conditions where perturbations are introduced to the microbial community of interest, and (iii) contain information on absolute microbial abundances.

Time-course data are essential for validating in silico model predictions. However, the collection of such data for the skin microbiota is often faced with practical challenges, including the susceptibility to contamination and biological noise (Kong et al, 2017), logistical issues with sampling (especially in studies involving clinical trial participants) (Ruuskanen et, 2022), and the need to maintain in vitro culture models under biologically relevant conditions (eg, a stable microbial composition) for extended periods (Boxberger et al, 2021). Moreover, such data collection procedures can be costly and time intensive, and may not appear to present immediate benefits to study design and wet-laboratory experiments.

Despite these challenges, time-course data are crucial for comprehending microbial interactions and community dynamics to advance skin microbiota research. Without time-course data, we may struggle to differentiate in silico model parameter sets that represent different dynamic trajectories but yield identical steady-state microbiota composition profiles, hindering our ability to infer the underlying microbial interaction network. Furthermore, collecting time-course data from the same individuals is crucial in disease conditions such as AD, which are multifactorial and exhibit significant interindividual variability in disease progression and phenotypic manifestations (Guttman-Yassky et al, 2018; Langan et al, 2020). Unlike cross-sectional data that provide only a snapshot of the disease trajectory, repeated longitudinal sampling can allow for a better understanding of intraindividual fluctuations over time and provide higher resolutions of microbiota dynamics for different disease phenotypes.

Appropriate perturbations (eg, through tape stripping or antibiotics) should also be introduced during data collection to obtain sufficiently significant dynamic changes in the microbiota profile for model parametrization, because the skin microbiota has been reported to be relatively stable over time, even under its constant exposure to the external environment (Oh et al, 2016). The necessity of time-course data for a microbial community under perturbation or evolution in inferring the microbiota interaction network is highlighted in several in silico gut microbiota studies. In those studies, Stein et al (2013) collected up to 12 cecal samples over 30 days from murine models, whereas Rao et al (2021) obtained nearly daily stool samples from 13 newborns during their first 6 weeks of life.

Finally, absolute abundance values, whether obtained from experimental measurements of total biomass or estimated by computational methods, are preferred to relative abundance data for drawing inferences about microbial interactions. A shift in the proportion of a single species does not indicate a change in its absolute abundance unless the total size of the microbial population remains constant, which is seldom true in microbial cultures and growth experiments, making longitudinal relative abundance data of little value. Although universal 16S ribosomal RNA qPCR is a widely used experimental approach for measuring absolute cell counts, it has been reported to exhibit large coefficients of variation across technical replicates, alongside biological noise arising from the wide ranges of 16S gene copy numbers in species (Smith et al, 2006; van de Velde et al, 2023). Flow cytometry offers rapid measurements of cell density and bacterial quantification (Wang et al, 2021); however, it may still face difficulties with detection limits owing to the challenges of transferring intact cells from skin swabs to solution, particularly for skin microbiota samples with low microbial loads (Morton et al, 2019). To overcome existing technical caveats in experiments, computational approaches such as BEEM (Li et al, 2019) have been recently developed to estimate total biomass values and infer in silico model parameters from relative abundance data. Nonetheless, empirical measurements of absolute abundances are still important in supporting and validating that computational biomass estimates can provide viable and realistic approximations.

### Moving toward a systems-level understanding of host-microbiota interactions in the skin

**Figure 2:**
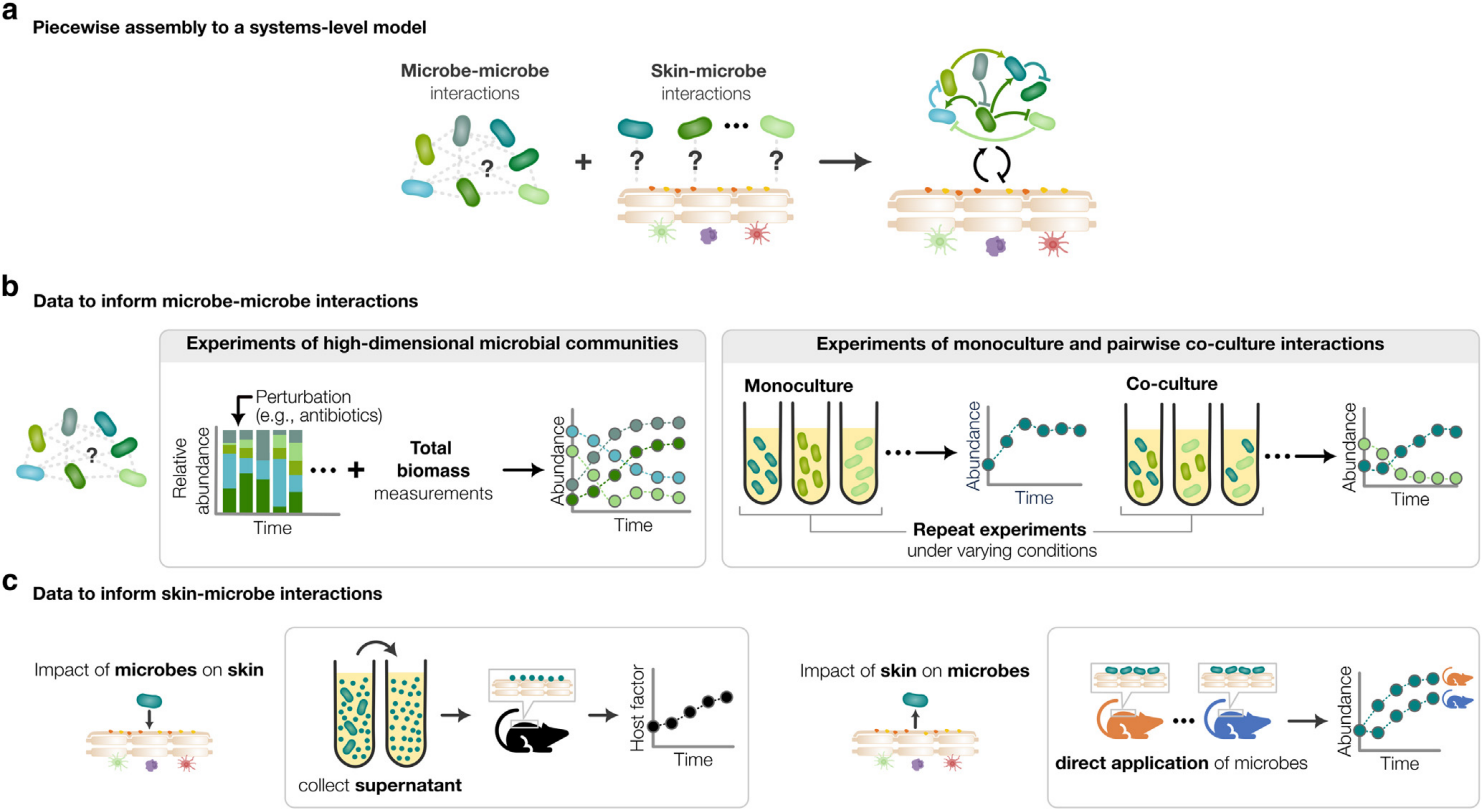
**Proposed approaches to collecting time-course experimental data for in silico skin microbiota modeling.** (**a**) Building up to a systems-level model of the skin microbiota through submodels of microbe-microbe and skin-microbe interactions. (**b**, **c**) Time-course data that can be used to respectively build and validate in silico models of microbe-microbe and skin-microbe interactions.

We next propose 2 constitutive themes that would benefit from in silico modeling, namely (i) microbe-microbe interactions and (ii) skin-microbe interactions (Figure 2). The union of these 2 themes should give a holistic view of the skin microbiota (Figure 2a), which would be difficult to achieve and assemble through wet-laboratory experiments alone. In this section, we discuss how empirical data collection and validation approaches for in silico gut microbiota models can be adapted to tackle each of these research themes (Figure 2b and c). Details of the experimental approaches proposed are listed in Table 4.

**Table 4:** Proposed Experiments for Empirical Data Collection and Prediction Validation of In Silico Skin Microbiota Models

Theme	Experiment Type	Assay Type	Method Details	Measurements Taken	What Predictions Would the Experiment Help to Validate?
Microbe-microbe	In vivo	Human skin sampling	• Collect clinical skin swabs from individuals over time	Sequencing counts and total microbial biomass	Microbial interactions and community dynamics
	In vitro	Synthetic microbial community	• Culture in media representative of human conditions • Collect samples from cultures over time	Sequencing counts and total microbial biomass	Microbial interactions and community dynamics
		Monoculture, coculture experiments	• Culture in growth media • Collect samples from cultures over time	Sequencing counts and total microbial biomass	Individual growth rates and pairwise interactions of microbes
				Metabolite concentrations using high-performance liquid chromatography	Metabolite dynamics and chemically mediated interactions between microbes
Skin-microbe	In vivo	Murine experiments	• Apply supernatants from microbial cultures onto mouse skin compromised by tape stripping	Transepidermal water loss (Almoughrabie et al, 2023) or skin impedance (Rinaldi et al, 2021)	Effects of microbes on skin barrier function
			• Apply microbial monocultures at specific initial densities (eg, 10^6^ CFU/cm^2^) onto mouse skin of varying physiologies • Collect skin swabs over time	Sequencing counts and total microbial biomass	Effect of skin barrier on microbial populations
	In vitro	3D organotypic skin models	• Apply single microbe or microbial communities • Can be supplemented with immune cells to mimic host immune responses • Collect skin swabs over time	Sequencing counts and total microbial biomass	Effect of host immune responses and skin physiologies on microbial interactions and community dynamics
			• Apply single microbe or microbial communities • Can be supplemented with immune cells to mimic host immune responses • Collect skin biopsy samples over time (Bolle et al, 2020; Rikken et al, 2023)	Histological examination and gram staining	Effect of microbes on skin physiology and bacterial load-induced tissue damage
		Suspension cultures	• Apply supernatants of microbial monocultures to normal human epidermal keratinocytes (Lai et al, 2010)	Skin AMP concentrations using ELISAs	Effects of microbes on skin AMP concentrations
			• Inoculate monocultures of key skin microbes independently with varying concentrations of skin AMPs	Sequencing counts and total microbial biomass	Effects of skin AMP concentrations on growth rates of microbes

Abbreviations: 3D, 3-dimensional; AMP, antimicrobial peptide; CFU, colony forming unit.

### Collecting time-course data of microbe-microbe interactions

Microbial interactions involve biochemical processes such as competition for resources, metabolite exchange, and antibiotic production targeting specific species (Foster and Bell, 2012; Ghoul and Mitri, 2016; Granato et al, 2019). Interactions can be aggregated into in silico model parameters representing growth influence, where their magnitudes indicate the strength of one microbe’s growth facilitation or hindrance on another. Collecting time-course data on microbial interactions and fitting it to in silico models enhances our understanding of key processes and mechanisms affecting growth, community behavior, and microbiota dynamics under perturbations.

#### In vivo methods

In vivo human sampling methods may offer the closest resemblance to microbial interactions occurring in the native human skin environment, but they also come with unique challenges. For example, the sampling procedures should be standardized and comparable (eg, swab samples collected from the same body site, surface area, and with consistent pressure applied) to minimize nonbiological noise (Kong et al, 2017). Human skin is also constantly exposed to the environment and has a lower biomass density than the gut (Belkaid and Segre, 2014), making its microbiota data more susceptible to contamination (Kong et al, 2017).

#### In vitro methods

In vitro cultures help to study microbial interactions under controlled conditions, but a key challenge lies in defining media compositions representative of the human skin and relevant disease conditions. Recent efforts in this area include the development of artificial sweat and sebum media (Swaney et al, 2023) and the optimization of media conditions to support a diverse synthetic community of skin microbes (Connors et al, 2023; Lekbua et al, 2024).

In vitro experiments can also be conducted through a bottom-up approach using monoculture and coculture experiments. Monoculture and coculture data are critical for understanding systems-level dynamics, as shown by Venturelli et al (2018). They found that these data types are more important than higher-order interactions for developing in silico models that effectively predict higher-dimensional microbial community dynamics.

### Collecting time-course data of skin-microbe interactions

The skin's functional elements include physical (corneocytes), chemical (AMPs from keratinocytes), and immunological (T cells in the epidermis) barriers that defend against infections (Elias, 2005, 2007). Although these skin components can shape the growth of skin microbes and the composition of the microbiota, microbes can also influence host responses (Belkaid and Segre, 2014; Byrd et al, 2018; Flowers and Grice, 2020). It is important to consider the bidirectional relationship between skin components and microbes, in addition to the interactions among microbes themselves, for a comprehensive understanding of skin microbiota dynamics.

#### In vivo methods

Murine models are frequently employed as in vivo models for studying skin-microbe interactions (Almoughrabie et al, 2023; Burian et al, 2017; Khadka et al, 2024; Zheng et al, 2022). However, despite their widespread use, empirical findings must be interpreted with caution owing to innate differences in mice and human skin physiologies (eg, reduced skin thickness and differences in immune responses) (Emmert et al, 2020). These physiological differences have driven the development of in vitro experimental platforms, including 3-dimensional organotypic skin models (eg, human skin equivalents [HSEs], human epidermal equivalents, and skin-on-a-chip models). They are typically developed with human skin cell lines or biopsies to replicate the native human skin conditions as closely as possible (Cho et al, 2024; Rikken et al, 2023). For example, Kamsteeg et al (2011) developed HSE models that mimic AD conditions using cells from healthy donors or patients with AD and supplementing the organotypic skin models with T helper 2 cytokines, IL-4 and IL-13, whereas van den Bogaard et al (2014) reported the development of an HSE model with keratinocytes and T cells. We envision that applying microbes and microbial communities to these platforms, such as inoculating SA onto HSEs to study tissue damage and driveline infections with percutaneous devices (Bolle et al, 2020), will greatly facilitate the exploration of host-microbiota interactions under human skin-like conditions.

#### In vitro methods

Traditional in vitro test-tube experiments—which offer the advantages of being well-established, repeatable, and high throughput—can also be performed to investigate skin-microbe interactions. Although these experiments usually occur in suspension cultures, which differ significantly from the solid, heterogeneous, and spatially limited environment of the skin, their simplicity and repeatability make them highly appropriate for conducting screening experiments across various combinatorial culturing conditions.

### Concluding remarks

In silico skin microbiota models offer a promising approach to systematically elucidate biological mechanisms of host-microbiota interactions and tackle the challenges of complex community dynamics when translating experimental studies to the microbiota level. However, the potential clinical utility of current in silico skin microbiota models is limited by the lack of fit-for-purpose empirical data and validation experiments. In silico skin microbiota models can be iteratively refined to achieve improved predictive performance and applicability in clinical settings through tailored experiments such as time-course sampling from skin and microbial cultures to study microbe-microbe interactions and applying microbial cultures and supernatants to murine models, 3-dimensional organotypic skin models, and human skin cell lines to study host-microbe interactions. We envision that validated in silico models with enhanced predictive power can help realise synergistic effects of experimental and computational approaches, advance our understanding of the role of the skin microbiota in health and disease, and guide the rational design of therapies.

## Materials and Methods

We searched the PubMed database on October 15, 2024 with no restriction on the starting month or year through to October 2024. We used the following string of terms to identify in silico models of the skin microbiota: (skin) AND (microbiota OR microbiome OR microbe OR microbial) AND (computational OR “*in silico*” OR theory OR mathematical) AND (model). To understand how in silico models of the skin microbiota compared with those of the gut microbiota, we also performed a literature search to identify in silico models of the gut microbiota using the following terms: gut AND (microbiota OR microbiome OR microbe OR microbial) AND (computational OR “*in silico*” OR theory OR mathematical) AND (model) AND (community OR consortia). All terms had to be present in either the title or abstract. The additional constraint of “community OR consortia” was added in our search for in silico gut microbiota models because we are interested in working toward developing microbial community models involving larger numbers of microbes.

We identified studies relevant to our analysis by applying the following inclusion and exclusion criteria:

• Inclusion: The study focuses on developing an in silico mechanistic model of microbe-microbe or host-microbe interactions within the skin or gut. The “host” includes any quantitative representation of the host environment (eg, resource pool or spatial effects) or host cells (eg, epithelial cells) through either a model parameter or variable.
• Exclusion:
  o Studies that are not original research articles (eg, opinion papers, review papers, textbooks);
  o Studies that were preprints at the time of the search;
  o Studies that focus on the development of toolboxes without examples of applications to the skin or gut microbiota; and
  o Studies of the skin or gut microbiota of other organisms or the environment, with no clear translation to human contexts (eg, honey bee gut microbiota).

After applying the inclusion and exclusion criteria, the search returned 3 and 38 papers for the skin and gut microbiota, respectively. We identified additional studies that were not returned by the search terms on the basis of our knowledge of the field (eg, through reading relevant references within papers). In total, we reviewed 6 in silico skin and 55 in silico gut microbiota studies.

## ORCIDs

Jamie Lee: http://orcid.org/0000-0001-8314-6651

Hiu Lam Athena Wu: http://orcid.org/0009-0007-8367-0243

Ahmad A. Mannan: http://orcid.org/0000-0001-7628-8416

Yuumi Nakamura: http://orcid.org/0000-0001-6256-8302

Masayuki Amagai: http://orcid.org/0000-0003-3314-7052

Alan D. Irvine: http://orcid.org/0000-0002-9048-2044

Reiko J. Tanaka: http://orcid.org/0000-0002-0769-9382

## Conflict of Interest

ADI reports personal fees from Sanofi Regeneron, AbbVie, Eli Lilly, Pfizer, UCB Pharma, Novartis, Dermavant, Benevolent AI, Menlo Therapeutics, Chugai, LEO Pharma, and Arena during the conduct of the study. The remaining authors state no conflict of interest.
